# A retrospective analysis of the clinicopathological characteristics of large cell carcinoma of the lung

**DOI:** 10.3892/etm.2014.2075

**Published:** 2014-11-17

**Authors:** RUI LIANG, TIAN-XING CHEN, ZHI-QIANG WANG, KE-WEI JIN, LIAN-YU ZHANG, QING-NA YAN, HUI-HUA ZHANG, WAN-PU WANG

**Affiliations:** 1 Department of Pathology, The First People’s Hospital of Yunnan Province, Kunming, Yunnan 650032, P.R. China; 2 Department of Oncology, The First Affiliated Hospital of Kunming Medical University, Kunming, Yunnan 650032, P.R. China; 3 Department of Pathology, Kunming Medical University, Kunming, Yunnan 650031, P.R. China; 4 Department of Pathology, The Affiliated Cancer Hospital of Tianjin Medical University, Tianjin 300060, P.R. China

**Keywords:** large cell carcinoma, lung cancer, pathology, retrospective analysis

## Abstract

The aim of the present study was to analyze and summarize the clinicopathological characteristics of large-cell lung carcinoma (LCLC) of the lung, in order to improve the definite diagnosis rate of LCLC. Clinicopathological data of 174 patients with LCLC, confirmed pathologically, were retrospectively reviewed. The 174 cases of LCLC accounted for 5.7% of the total lung cancer cases during the corresponding time period at the Affiliated Cancer Hospital of Tianjin Medical University (Tianjin, China), among which there were 131 males and 43 females with an average age of 61.4 years. The postoperative pathological diagnosis of the 174 cases showed 80 cases of classic LCLC, 64 cases of large cell neuroendocrine carcinoma (LCNEC), six cases of combined LCNEC, 19 cases of basaloid carcinoma, three cases of clear cell carcinoma and two cases of lymphoepithelioma-like carcinoma. Of the total 174 LCLC cases, 96 patients exhibited lymph node metastasis. LCLC is a highly aggressive malignancy with a high tendency of invasion and metastasis, although the incidence rate is low. A definite diagnosis of LCLC primarily relies on the pathological diagnosis. Each subtype of LCLC has its own pathomorphological and immunohistochemical characteristics.

## Introduction

Lung cancer is one of the most common malignancies worldwide, of which the incidence rate and mortality rank are the highest amongst the different malignancies (1). The main histological types include squamous cell carcinoma, adenocarcinoma, large-cell lung carcinoma (LCLC) and small-cell lung carcinoma (SCLC). LCLC is a type of undifferentiated carcinoma, that lacks the cell differentiation and structural characteristics of small-cell lung cancer, adenocarcinoma and squamous cell carcinoma. The incidence rate of LCLC is significantly lower compared with the other types, with LCLC accounting for ~9% of total lung cancer cases, as reported by the World Health Organization (WHO) (2). However, in previous studies by Hanagiri *et al* (3) and Battafarano *et al* (4), the incidence rate was reported as 5.8% (57/975) and 3.9% (82/2,099), respectively. In addition, the incidence rate in China has been reported to be between 1 and 2% (5,6). The clinical manifestation of LCLC is not typical, and the early diagnosis rate is low. LCLC is prone to regional lymph node and distant metastasis, with poor prognosis. It is not sensitive to chemotherapy and there is no standard treatment schedule. Due to the high malignancy, LCLC has been increasingly studied in recent years (7–11). The lower incidence of LCLC in China has been hypothesized to be the result of geographic or ethnic differences. However, with improvement in the diagnostic levels of Chinese pathologists on the morphological features and immunohistochemical characteristics of LCLC, the definite diagnosis rate of LCLC has been significantly improved. In the present study, the clinicopathological data from 174 patients with LCLC were retrospectively analyzed, with the aim of summarizing the specific clinicopathological features of LCLC and thereby improving the definite diagnosis rate of LCLC.

## Materials and methods

### Patients and tumor specimens

A total of 174 patients with LCLC (male, 131; female, 43; gender ratio, 3.05:1; mean age, 61.4 years; age range, 28–82 years), pathologically confirmed at the Department of Pathology at the Affiliated Cancer Hospital of Tianjin Medical University (Tianjin, China), were retrospectively reviewed between 2012 and 2013. The incidence of LCLC accounted for 5.7% of the total lung cancer cases (n=3,053) during the corresponding time period. A total of 125 patients (71.8%) had a history of smoking, with an average smoking index of 670. This value is calculated by the number of cigarettes smoked per day × years of smoking and divided by 125 (12). The clinical symptoms included a cough and expectoration (n=128, 73.6%), bloody sputum or hemoptysis (n=88, 50.6%), chest pain (n=61, 35.1%), dyspnea and short breath (n=34, 19.5%) and a fever (n=27, 15.5%). Tumor, node and metastasis (TNM) classification of the cancer tissues revealed 23 cases of stage Ia, 42 cases of stage Ib, 10 cases of stage IIa, 46 cases of stage IIb, seven cases of stage IIIa, 37 cases of stage IIIb and nine cases of stage IV. All the patients had intact clinicopathological data. The study was conducted in accordance with the Declaration of Helsinki, and with approval from the Ethics Committee of the Affiliated Cancer Hospital of Tianjin Medical University. Written informed consent was obtained from all the participants.

### Imaging examination

All patients underwent a chest X-ray (DR-F; General Electric Corp., Fairfield, CT, USA) or computed tomography (CT) scan (Optima CT660; General Electric Corp.), which showed a single lesion in each patient. The maximum diameter of the tumors varied between 1.5 and 11.5 cm, with an average of 4.7 cm. The majority of the tumors were of a peripheral type (89.1%, 155/174), with the central type accounting for only 10.9% (19/174). In total, 101 cases were located in the right lung (superior lobe, 53; middle lobe, 8; lower lobe, 29; near the hilum of the lung, 11) and 73 cases were located in the left lung (superior lobe, 47; lower lobe, 18; near the hilum of the lung, 8). The imaging examination revealed lobulation in 105 cases (60.3%), a burr shadow in 53 cases (30.5%), pleural effusion in 10 cases (5.7%), atelectasis in 12 cases (6.9%) and mediastinal lymphadenectasis in 34 cases (19.5%).

### Histological examination

Prior to surgery, the 174 patients underwent a sputum exfoliative cytopathological examination. In addition, 69 cases received a fiber bronchoscope biopsy, 21 cases received a CT-guided lung puncture biopsy and 37 patients received a supraclavicular or cervical lymph node biopsy. All of the biopsy tissues were sent for routine histopathological examination. They were fixed in 10% neutral formalin and desiccated and embedded in paraffin, and then were conventionally sectioned and stained with hematoxylin and eosin. Following surgery, all of the tissues were sent for routine histopathological examination and immunohistochemical stains (13). Specific antibodies including mouse anti human cytokeratin 5/6 (CK5/6), high molecular weight cytokeratin (HCK; 34βE12), P63 (4A4), thyroid transcription factor 1 (TTF 1; SPT24), cytokeratin 7 (CK7; OV-TL 12/30)monoclonal antibodies, and rabbit anti human synaptophysin (Syn; SP11) and chromogranin A (CgA; SP12) were purchased from Fuzhou Maixin Biological Technology Co., Ltd. (Fuzhou, China). Polymeric horseradish peroxidase conjugated anti-mouse/rabbit IgG (Fuzhou Maixin Biological Technology Co., Ltd.) was used as the secondary antibody.

### Treatment

Of the 174 patients, 137 cases received surgical treatment, including exploratory thoracotomy (n=9), regional wedge excision (n=11), pulmonary lobectomy (n=98) and total pneumonectomy (n=19). Among them, 73 patients also received postoperative chemotherapy, 12 patients received postoperative radiotherapy and 13 patients received postoperative biotherapy. A total of 32 patients undertook palliative therapy, including simple chemotherapy (n=19), radiotherapy (n=5) and biotherapy (n=8). In total, 5 patients gave up treatment following confirmation of LCLC by biopsy of the lymph nodes.

### Statistical analysis

All data were analyzed using the SPSS 13.0 software program (SPSS, Inc., Chicago, IL, USA). The two- or multi-sample comparison of the survival rate was performed using chi-squared test. For all statistical analysis, P<0.05 was considered to indicate a statistically significant difference.

## Results

### Preoperative cellular and histological examination

Sputum exfoliative cytological examinations of the 174 patients revealed only two cases with adenocarcinoma cells and five cases with poorly differentiated cancer cells. Among the 69 patients that underwent a fiber bronchoscope biopsy, three cases were diagnosed as LCLC, one case was diagnosed as combined SCLC, two cases were diagnosed as squamous cell carcinoma and three cases were diagnosed as poorly differentiated carcinoma (histological type was uncertain). In addition, among the 21 cases that received a simultaneous lung puncture biopsy, there were four cases of LCLC supported by immunohistochemistry, one case of neuroendocrine carcinoma and three cases of poorly differentiated carcinoma (histological type was uncertain).

### Postoperative histological examination

**Figure 1:**
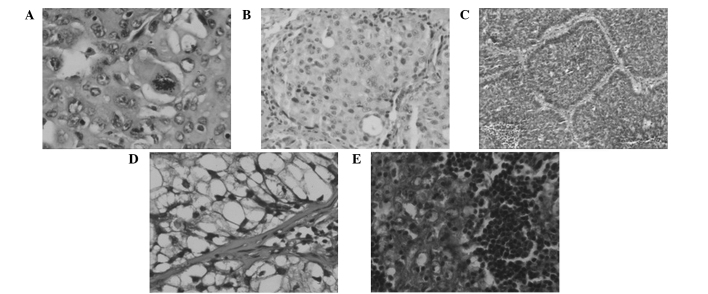
Morphology of the different subtypes of LCC (hematoxylin and eosin stain). (A) Classical LCC (magnification, ×100); (B) large cell neuroendocrine carcinoma (magnification, ×40); (C) basaloid carcinoma (magnification, ×40); (D) clear cell carcinoma (magnification, ×100); and (E) lymphoepithelioma-like carcinoma (magnification, ×100). LCC, large cell carcinoma.

All the specimens were reviewed by two experienced pathologists from the Affiliated Cancer Hospital of Tianjin Medical University, according to the revised histological classification standards of lung cancer for the diagnosis and classification of LCLCs established by the WHO (third edition) (2). All the 174 patients (including 37 cases that underwent supraclavicular or cervical lymph node biopsy) were diagnosed as LCLC. The cases were classified into classic LCLC (46.0%, 80/174; Fig. 1A), large cell neuroendocrine carcinoma (LCNEC; 36.8%, 64/174; Fig. 1B), complex LCNEC (3.4%, 6/174), basaloid (10.9%, 19/174; Fig. 1C), clear cell (1.7%, 3/174; Fig. 1D) and lymphoepithelioma-like carcinomas (1.1%, 2/174; Fig. 1E). In addition, 96 cases were confirmed pathologically to be lymph node positive and were located at the hilum of the lung, parabronchus, supraclavicular, cervical and mediastinal sections, accounting for 84.2% of the 114 patients that underwent a lymph node biopsy.

### Postoperative immunohistochemical examination

Routine immunohistochemical analysis was performed on the 174 specimens. In the 80 cases of classic LCLC, the positive expression rates of 34βE12, CK5/6 and P63 were 100, 76.3 and 52.5%, respectively; the positive expression rates of CK7 and TTF-1 were 100 and 51.3%, respectively; however, positive expression of Syn and CgA was not observed. Among the 64 cases of LCNEC, the positive expression rates of Syn and CgA were 85.9% (diffusely positive, 78.1%; focally positive, 7.8%) and 68.8% (diffusely positive, 46.9%; focally positive, 21.9%), respectively. Furthermore, positive expression of Syn or CgA was observed in each patient (100%), with 39 cases (60.9%) exhibiting positive expression for both neuroendocrine markers. The positive expression rates of 34βE12, CK5/6 and P63 in the LCNEC tissues were 35.9, 45.3 and 25%, respectively, with 54.7% of the LCNEC tissues positive for at least one squamous epithelial marker. The positive expression rates of CK7 and TTF-1 were 84.4 and 65.6%, respectively, and positive expression of at least one glandular epithelial marker was observed in 54 cases (84.4%). Within the 19 cases of basaloid carcinoma, the positive expression rates of 34βE12, CK5/6 and P63 were 78.9, 68.4 and 52.6%, respectively. All the basaloid carcinoma tissues were positive for at least one of these markers (100%). CK7 was expressed in all the basaloid carcinoma tissues; however, TTF-1 or CgA expression was not observed. Syn was weakly expressed in the basaloid carcinoma tissues, accounting for 5.3% of cases.

### Follow-up examination

Among the 174 patients with LCLC, 83 cases diagnosed in 2012 were followed-up for one year; however, five cases did not complete the follow-up (Table I). The one-year survival rate of the cases was 57.8% (48/83). No statistically significant association was identified between the survival rate and the patient gender, age, tumor location and tumor size. However, the survival rate of the patients was found to be significantly correlated with the TNM stage, N stage, M stage, occurrence of radical resection and pathological subtype. The N stage was used to decide whether there is regional lymph node metastasis and the metastasis degree; M stage was used to decide whether there is distant metastasis. The one-year survival rate of the patients classified with stage I was significantly higher compared with the patients classified with a higher stage (P<0.05). With regard to the N stage, the one-year survival rate was significantly higher in patients with stage N_0_ than in patients with other stages (P<0.05), and was also higher in patients with stage N_1_ than in patients with stage N_3_ (P<0.001). Patients classified as stage M_0_ also had a significantly higher survival rate compared with patients classified as stage M_1_ (P=0.016). Patients that underwent a radical resection had a significantly higher survival rate than patients who received palliative treatment (P=0.018). In addition, the survival rate of the patients with classic LCLC was significantly higher compared with the patients with LCNEC (P=0.003). The additional 91 patients were diagnosed in 2013 and were followed-up for less than one year. To date, three cases have been lost and there have been 13 cases of mortality.

## Discussion

The incidence rate of LCLC is significantly lower than other types of lung cancer, including squamous cell carcinoma, adenocarcinoma and SCLC. With preliminary statistics, 174 patients were diagnosed with LCLC at the Department of Pathology of the Affiliated Cancer Hospital of Tianjin Medical University between 2012 and 2013, accounting for 5.7% of the total lung cancer cases (3,053 cases) during the corresponding time period. Improvements to the pathological diagnostic level of LCLC and the development of immunohistochemical techniques have significantly increased the definite diagnosis rate of LCLC (14–17). In the present study, routine immunohistochemistry was performed on the LCLC tissues, including three squamous cell markers (CK5/6, 34βE12 and P63), two glandular epithelial markers (TTF-1 and CK7) and two neuroendocrine markers (Syn and CgA). Barbareschi *et al* (18) diagnosed and classified LCLC via the detection of the squamous markers, P63, CK5 and desmocollin 3, the glandular epithelial markers, TTF-1, Napsin A and CK7, and the neuroendocrine markers, Syn, CgA and CD56.

Since LCLCs are predominantly located in the peripheral area of the lungs, there were fewer positive results from preoperative sputum exfoliative cytology and bronchofiberscope biopsy examinations. In addition, due to small specimen, tissue extrusion and deformation it is difficult to be used for immunohistochemistry. Therefore the preoperative diagnosis rate of LCLC is far lower compared with that of squamous cell carcinoma, SCLC and adenocarcinoma, whereas CT-guided lung puncture biopsy has a relatively high positivity rate. In the present study, only seven cases (4.0%) were confirmed or considered to be LCLC using preoperative sputum exfoliative cytology, bronchofiberscope biopsy or lung puncture biopsy. Comparatively, the positive ratio of the lung puncture biopsy was superior to that of the other two methods, which is consistent with the results reported by Watanabe *et al* (19). Doddoli *et al* (20) reported only one out of 20 cases of LCLC as definitely diagnosed using a preoperative bronchofiberscope biopsy.

Clinically, LCLC is difficult to be differentiated from other types of lung cancer. Diagnosis primarily depends on histopathological examination, with the aim to exclude squamous cell carcinoma, adenocarcinoma and SCLC. Currently, the histopathological diagnosis of classic LCLC is based on the following evidence (2). Firstly, the tumor cells are bulky and polygonal. Secondly, tumor cells have a moderate amount of cytoplasm. Thirdly, the tumor cells present large nuclei, vesicular nuclei and prominent nucleoli. Fourthly, the tumor cells are nests. Fifthly, the ultrastructure of the tumor cells exhibits a small amount of adenoid or squamous differentiation. In the present study, classic LCLC accounted for 46.0% of the LCLC cases (80/174). Classic LCLC should be identified from poorly differentiated squamous cell carcinoma and the solid invasive adenocarcinoma. Generally, a small amount of cell keratinization or intercellular bridges can be observed in poorly differentiated squamous cell carcinoma, while mucus droplets in the cytoplasm can be observed in the solid invasive adenocarcinoma. As detected using an immunohistochemical method, LCLCs express varying degrees of squamous and glandular epithelial markers, but no neuroendocrine markers. All the 80 cases of classic LCLC were in compliance with the aforementioned morphological and immunohistochemical characteristics.

According to the third edition of the histological classification criteria for lung cancer established by the WHO (2), in addition to classical LCLC, LCLC also can be classified into the following six subtypes: LCNEC, complex LCNEC, basaloid carcinoma, clear cell carcinoma, lymphoepithelioma-like carcinoma and LCLC with striated muscle-like phenotype. LCNEC is the most common type, and can be diagnosed based on the following histological features (2,21,22): i) Large tumor cell volume; ii) moderate or rich amount of cytoplasm; iii) prominent nucleoli and mitotic count: >11 mitoses per 10 high-power fields (HPFs); iv) tumor cells exhibiting morphological features of neuroendocrine differentiation, including organ-like nests, trabecular, rosette and palisade arrangements; v) large areas of necrosis are commonly observed; and vi) immunohistochemical positive staining for at least one neuroendocrine marker. A total of 64 cases of LCNEC (36.8%) were identified in the current study, with Syn and CgA expression rates of 85.9 and 68.8%, respectively. Additionally, the expression rate, intensity and scope were higher for Syn than CgA, indicating that Syn is a more valuable individual neuroendocrine marker for the diagnosis of LCNEC. All the LCNEC cases exhibited positive expression for Syn or CgA, with 42 cases expressing Syn and CgA, 25 cases expressing Syn but no CgA, and 14 cases expressing CgA but no Syn, indicating that the expression of these two markers does not cover each other. Thus, a combination of these two neuroendocrine markers or more may improve the definite diagnosis rate of LCNEC. These observations were consistent with the results of Tanaka *et al* (23). The diagnosis of LCNEC should be differentiated from atypical carcinoids, as these are two types of neuroendocrine tumor that exhibit organ-like, trabecular, rosette and palisading morphological characteristics, as well as necrosis. However, atypical carcinoids exhibit relatively few mitotic figures (2–10/10 HPF) and LCNECs commonly show broader necrosis than atypical carcinoids. In addition, LCNEC cases should be differentiated from basaloid carcinoma.

The histopathological characteristics of basaloid carcinomas are as follows (1,10): i) Tumor cells are relatively small with cube to fusiform shape, appearing monomorphic; ii) decreased levels of cytoplasm; iii) moderate nuclear chromatin, finely granular, a lack of nucleoli or little punctiform nucleoli, and mitotic figures of ≥15/10 HPF; iv) tumor cells presenting as solid nests or anastomotic beam-like ordering, around which is a palisade arrangement, with rosettes found in 1/3 of cases; v) comedonecrosis is commonly observed; vi) ultrastructure lacks squamous differentiation; and vii) immunohistochemical analyses are often positive for hemopoietic cell kinase (HCK) and negative for TTF-1 and neuroendocrine indicators. The 19 cases of basaloid carcinoma in the present study accounted for 10.9% of the total LCLC cases. Basaloid carcinomas should firstly be differentiated from LCNECs, since these two tumors exhibit necrosis, a palisade arrangement and occasionally rosettes. However, the majority of LCNECs present with large extensive necrosis, while basaloid carcinomas often exhibit comedo-like necrosis. Immunohistochemical staining is of great importance for the identification of these two types. All the LCNECs diffusely expressed at least one of the neuroendocrine indicators; however, the majority of basaloid carcinomas did not express neuroendocrine indicators, with only 10% focally expressing one neuroendocrine indicator. In addition, HCK was not expressed in LCNEC cases, but often expressed in basaloid carcinoma. Approximately 50% of the LCNECs expressed TTF-1, but no basaloid carcinoma expressed TTF-1. Basaloid carcinoma should be also differentiated from the basal-like subtype of squamous cell carcinoma and SCLC.

Combined LCNEC refers to tumors with morphological and immunohistochemical characteristics of LCNEC, as well as characteristics of well-defined non-small cell lung cancer, including squamous cell carcinoma, adenocarcinoma, giant cell carcinoma and spindle cell carcinoma. LCNEC combined with SCLC has been classified into the SCLC category. In the present study, there were only six cases of combined LCNEC, five of which were combined with invasive adenocarcinoma and one that was combined with squamous cell carcinoma. Morbini and Inghilleri (24) reported a novel type of combined LCNEC, in which sections of the tumor exhibited morphological and immunohistochemical characteristics of LCNEC, while other sections were characteristic of basaloid carcinoma.

Histologically, clear cell carcinomas have large tumor cells in a polygonal shape, abundant cytoplasm and watery transparency or foam. The tumor cells exhibit diffuse and lamellar growth, and the ultrastructure lacks adenoid or squamous differentiation. Clinically, metastatic clear cell carcinomas from kidney, thyroid and the salivary gland should be excluded from the diagnosis of primary lung clear cell carcinoma. In the present study, there were only 3 cases of lung clear cell carcinoma, which were clinically excluded from the metastatic carcinomas from other tissues metastasis. In addition, only two cases of lymphoepithelioma-like carcinoma were identified, exhibiting a large volume of tumor cells, syncytium-like, large nuclei, vesicular nuclei and evident red nucleoli. The tumor cells exhibited diffuse and lamellar growth with a clear pushing boundary. Lymphatic infiltration was also observed. Diagnosis of LCLC combined with a skeletal muscle-like phenotype requires ≥10% of the tumor cells to be composed of muscle-like cells, whose cytoplasm contains an eosinophilic body. This subtype is very rare, and no cases were observed in the present study.

Currently, the treatment of LCLC is a comprehensive, primarily with surgical excision (3,6,25). In the present study, the radical resection rate of LCLC was 73.5% (128/174), of which the majority underwent a lobectomy. Whether chemotherapy is required following surgery remains controversial (26). Although biological treatment has achieved good results in the treatment of a variety of solid tumors (27,28), there have been no specific studies on the treatment efficacy of biological therapy in patients with LCLC. The 83 patients diagnosed with LCLC in 2012 were followed-up for one year, and the one-year survival rate of patients treated with radical resection (65.1%) was significantly higher compared with that of patients treated with palliative treatment (35.0%). No statistically significant difference was observed in the one-year survival rate of patients receiving postoperative radiochemotherapy and patients that were treated with surgery alone. Hanagiri *et al* (3) reported that the postoperative five-year survival rate of Japanese patients with LCLC of the lung receiving surgery was 60.5%, which was significantly higher compared with that of non-surgical patients. In addition, Saji *et al* (29) demonstrated that the five-year survival rate of patients with LCLC receiving comprehensive treatment was superior to that of patients treated with surgery alone. However, this may have resulted from the short follow-up period in this study.

In addition to radical resection, the present study found that the TNM, N and M stages and histological types were significantly correlated with the one-year survival rate of the 83 patients with LCLC confirmed in 2012. The one-year survival rate of the patients with stage I was significantly higher compared with the patients with stages II, III and IV. The survival rate was also significantly higher in patients with stage II when compared with patients with stages III and IV. Similarly, the one-year survival rate of patients with stage N_0_ was significantly higher than that of patients with stages N_1_, N_2_ and N_3_, and was also significantly higher in patients with stage N_1_ when compared with patients with stage N_3_. Furthermore, the one-year survival rate of the patients with stage M_0_ was significantly higher compared with the patients with stage M_1_. Among the various subtypes of LCLC, the patients with classic LCLC had a significantly higher one-year survival rate (74.4%) than patients with LCNEC (38.7%). Shimada *et al* (30) and Sun *et al* (11) also reported that among the subtypes of LCLC, LCNEC is more aggressive with a higher degree of malignancy, and clinical features that are closer to SCLC. Among the 91 patients diagnosed with LCLC in 2013, 13 patients succumbed during the follow-up period, indicating that LCLC of the lung is a highly malignant and aggressive tumor.

Future studies should expand the sample size and follow-up time period in order to summarize the morphological and immunohistochemical features of LCLC more accurately, analyze the impacting factors on patient prognosis more comprehensively and compare the pros and cons of the treatment programs in more detail. Only by following the diagnostic criteria of LCLC strictly and fully combining immunohistochemical analyses with electron microscopy can the diagnosis rate of LCLC and its subtypes be improved. Subsequently, a basis for the clinical standardization of the comprehensive treatment of LCLC may be established.

## Figures and Tables

**Table I:** Association between the one-year survival rate of 83 patients with LCC diagnosed in 2012 and their clinicopathological features.

Variable	One-year survival rate, % (n)
TNM stage	
I	82.9 (29/35)
II	56.0 (14/25)
III	26.3 (5/19)
IV	0 (0/4)
N stage	
N_0_	90.5 (19/21)
N_1_	63.4 (26/41)
N_2_	25 (1/4)
N_3_	11.8 (2/17)
M stage	
M_0_	60.8 (48/79)
M_1_	0 (0/4)
Treatment	
Radical resection	65.1 (41/63)
Palliative treatment	35.0 (7/20)
Pathological subtype	
Classic LCC	74.4 (29/39)
LCNEC	38.7 (12/31)
Basaloid carcinoma	50.0 (4/8)
Combined LCNEC	50.0 (1/2)
Clear cell carcinoma	50.0 (1/2)
Lymphoepithelioma-like carcinoma	100 (1/1)

TNM, tumor, node and metastasis; LCC, large cell carcinoma; LCNEC, large cell neuroendocrine carcinoma.
